# Application of hyperbolic geometry in link prediction of multiplex networks

**DOI:** 10.1038/s41598-019-49001-7

**Published:** 2019-08-30

**Authors:** Zeynab Samei, Mahdi Jalili

**Affiliations:** 1 Department of Computer Science, Institute for Research in Fundamental Sciences (IPM), Tehran, Iran; 2 School of Engineering, RMIT University, Melbourne, Australia

**Keywords:** Applied mathematics, Applied mathematics, Applied mathematics, Computer science, Applied mathematics

## Abstract

Recently multilayer networks are introduced to model real systems. In these models the individuals make connection in multiple layers. Transportation networks, biological systems and social networks are some examples of multilayer networks. There are various link prediction algorithms for single-layer networks and some of them have been recently extended to multilayer networks. In this manuscript, we propose a new link prediction algorithm for multiplex networks using two novel similarity metrics based on the hyperbolic distance of node pairs. We use the proposed methods to predict spurious and missing links in multiplex networks. Missing links are those links that may appear in the future evolution of the network, while spurious links are the existing connections that are unlikely to appear if the network is evolving normally. One may interpret spurious links as abnormal links in the network. We apply the proposed algorithm on real-world multiplex networks and the numerical simulations reveal its superiority than the state-of-the-art algorithms.

## Introduction

Many real biological, social and technological systems are modeled as networks in which nodes and links represent entities and different kinds of connections respectively. Network analysis in complex systems such as biology, ecology, computer science and sociology has become very important and applicable^1^. One of the major topics in network science is to predict missing, forthcoming and spurious links^2^. Many different link prediction algorithms have been introduced that use structure information of networks. Most of them are classified in similarity-based prediction methods, which work under the assumption that the probability of existing a link between two nodes is depended to their similarity^3^.

There are other types of link prediction methods such as Hierarchical Structure Model and Stochastic Block Model which are based on maximum likelihood analysis^2,4,5^. Recently the study of hyperbolic geometry based on the network structure has become useful in solving the link prediction problem. Considering the hyperbolic geometry of networks, HyperMap method was proposed by Papadopoulos *et al*.^6^. This method first map target networks into hyperbolic space, and then predict the missing links using the hyperbolic coordinates of node pairs^7^.

Recent studies^8–10^ have shown that many real network systems are modeled better in multiple layers to show different kinds of interactions between individuals^11,12^. Multiplex networks are a special kind of multilayer networks in which the number of nodes in all layers is the same. Some studies have shown that the structural features of different layers in multiplex networks are indeed correlated to each other^1,13^. So it can be supposed that considering the interlayer information can enhance the performance of link prediction in each layer of a multiplex network. In this paper, we investigate the node similarity index based on hyperbolic geometry and the layer relevance of the multiplex networks for predicting the spurious and missing links. In this method, we improve the performance of the link prediction based on hyperbolic distance considering both the popularity and similarity of nodes by combining the similarity indices in multiplex networks.

Using the interlayer information in solving the missing link prediction in multiplex networks has been considered before in a number of works. Pujari *et al*.^14^ used a decision tree classifier to predict the interaction of the coauthorship network in a multiplex collaboration network with three layers. Hristova *et al*.^15^ used a supervised classifier for link prediction in a two layer network containing Foursquare and Twitter using the interlayer information. In another work, Sharma *et al*.^16^ proposed a new method considering weight for each layer of multiplex network and used it to solve the link prediction problem in the target layer. Yao *et al*.^17^ proposed a novel method based on interlayer and intralayer information to solve the missing link prediction problem in multiplex networks. Hajibagheri *et al*.^18^ proposed a holistic method considering the information of all layers simultaneously in link prediction of a target layer in multiplex networks. Also, Guimerà *et al*. used Stochastic Block Models to predict missing and spurious links in noisy networks. Zeng *et al*.^19^ studied the impact of spurious link identification methods on distortion of networks’ structure and dynamics. In another study, Zhang *et al*.^20^ measured the inter-similarity using the local diffusion processes in bipartite networks. Samei *et al*.^21^ proposed a method to identify spurious links in multiplex networks. In fact, they proposed a method to employ interlayer information to improve the performance of spurious link prediction in the target layer.

In the context of hyperbolic geometry of network, Krioukov *et al*.^22^ introduced the mapping of networks to hyperbolic space. They used the underlying hyperbolic geometry of network to study the functionality and structure of complex networks. They showed that the strong clustering and the heterogeneous degree distribution are natural reflections of the negative curvature and other properties of the hyperbolic geometry of complex networks. Then, Papadopoulos *et al*.^23^ studied the impact of popularity and similarity in networks’ growth. They developed a framework to suggest that new connections can be made between node pairs with an optimized trade-off between popularity and similarity. In another work, Papadopoulos *et al*.^24^ presented the HyperMap method to map a network to its underlying hyperbolic space and used the hyperbolic distance as a similarity measure to solve the link prediction problem. Different from these works, other methods were introduced to infer hidden geometry of complex networks^6,25^. Recently, Muscoloni *et al*.^26,27^ introduced a nonuniform popularity-similarity optimization model (*N-PSO*). This model was used to predict the missing links using the community structure of the networks in *N-PSO* that improved the performance of the link predictors significantly. Muscoloni *et al*. also proposed an intelligent machine to infer the network hyperbolic geometry based on an “angular coalescence” phenomenon^28^. A minimum curvilinear automata has been recently proposed to embed hyperbolic geometry of networks and used it for link prediction^29^.

In this paper, our proposed similarity indices based on hyperbolic geometry of network benefit both intralayer and interlayer information to solve the spurious and missing link prediction in multiplex networks. Based on that the experimental results on four single layer synthetic networks and six real multiplex networks show that the performance has been improved when the hyperbolic-based methods is used and the node pairs similarity measures are computed considering both interlayer and interlayer information.

## Methods

Consider *G* = (*G*^1^, *G*^2^, …., *G*^*M*^) as a multiplex network with *N* nodes in each of *M* layers, where *G*^*α*^ = (*V*^*α*^, *E*^*α*^)represents the network of layer *α* with *V* as the set of nodes and *E* as the set of links^11,12,30^. We can assume ${A}^{[\alpha ]}=\{{a}_{ij}^{[\alpha ]}\}$ as the adjacency matrix of each layer *G*^*α*^, where for 1 ≤ *α* ≤ *M* and 1 ≤ *i*, *j* ≤ |*V*^*α*^|^31^:

1 $${a}_{ij}^{[\alpha ]}=\{\begin{array}{cc}1\, & if({v}_{i}^{[\alpha ]},\,{v}_{j}^{[\alpha ]})\in {E}^{\alpha }\\ 0 & otherwise\end{array}$$

In the context of unsupervised link prediction, many similarity measures are defined to find the likelihood of link existence between each node pair (*i*, *j*). In multiplex networks the similarity score in layer *α* is shown by ${s}_{ij}^{\alpha }$. After computing the similarity scores for all potential node pairs in each layer, a ranking method can be used to choose the top ranked pairs which have more chance to make a connection. The key issue is how to calculate the similarity scores based on the known topology of networks. Recent studies have shown that the structure of the layers in multiplex networks are mostly dependent^32,33^. Hence, one of the main challenges in solving the link prediction problem in multiplex networks is to find an appropriate similarity measure that can benefit the relevant information of all layers^30^. Based on this, here we use both the information of the target layer (intralayer information) and other layers (interlayer information) and combine them based on layer relevance to improve the performance of link prediction compared with the single-layer based methods.

In the case of missing link prediction, the goal is to estimate the probability of existence of non-observed links based on the current topology of network and available node’s features in network G(*V*, *E*). Since the missing links are not known, we assume that a fraction of observed links *E*, is missing and the goal of link prediction is to identify them. In order to do that, in each iteration a fraction of the observed links _*E*_, is removed based on k-fold decomposition method and the proposed methods are supposed to predict them. In the case of identifying spurious links, the task is to evaluate whether the observed links are reliable enough based on the current topology of the network. In order to do that, in each iteration, some nonexistent links are randomly added to the link set and the proposed methods are supposed to identify them^34^. Precision is used here to quantify the accuracy of a link prediction method which is defined as:

2 $$Precision=\frac{|TP|}{|TP|+|FP|}$$

where |*TP*| is the number of positive predictions that are truly predicted and |*FP*| is the number of positive predictions that are wrongly predicted.

### Node similarity index

Description of the similarity indices is given in the following.

#### Existing measures

- **Preferential Attachment (PA):** This index is based on the node degrees and for each node pair *i* and *j* is defined as:

3 $${s}_{ij}^{PA}=\Vert {{\rm{\Gamma }}}_{i}\Vert \times \Vert {{\rm{\Gamma }}}_{j}\Vert $$

where ||Γ_*i*_|| indicates the number of neighbors of node *i*.

- **Common Neighbors (CN)**: For each node pair *i* and *j*, this index counts the number of neighbors that are common between them and is defined based on the assumption that node pairs with more common neighbors are more likely to make connection. It is defined as:

4 $${s}_{ij}^{CN}=\Vert {{\rm{\Gamma }}}_{i}\cap {{\rm{\Gamma }}}_{j}\Vert $$

- **CAR:** This measure considers both common neighbors of each node pair and the number of connections between the common neighbors, and is computed as below:

5 $${{s}}_{{ij}}^{{CAR}}={{s}}_{{ij}}^{{CN}}\cdot {{s}}_{{ij}}^{{LCL}}$$

where ${s}_{ij}^{CN}$ is the number of common neighbors between (*i*, *j*) and ${s}_{ij}^{LCL}$ is the number of links between nodes in the common neighbors set^35^.

- **CJC:** This measure is a modified version of Jacard measure and is defined as below:

6 $${{s}}_{{ij}}^{{CJC}}=\frac{{{s}}_{{ij}}^{{CAR}}}{\parallel {{\Gamma }}_{{i}}\cup {{\Gamma }}_{{j}}\parallel \,}$$

where ${s}_{ij}^{CAR}$ is the similarity measure *CAR* defined above and $\Vert {{\rm{\Gamma }}}_{i}\cup {{\rm{\Gamma }}}_{j}\Vert$ is total number of neighbors of nodes *i* and *j*^35^.

- **Hyperbolic distance (HP):** This measure computes the hyperbolic distance (Eq. (8)) of each node pair *i* and *j* based on *Hypermap* method. *HyperMap* is based on Maximum Likelihood Estimation. It finds the radial and angular coordinates *r*_*i*_, *θ*_*i*_ for all nodes *i* ≤ *N*, which maximizes the likelihood:

7 $${L}=\prod _{1\le i < j\le N}{p}{({x}_{ij})}^{{\alpha }_{ij}}{[1-p({x}_{ij})]}^{1-{\alpha }_{ij}}$$

where the product is computed over all node pairs *i*, *j* and *x*_*ij*_ is defined as the hyperbolic distance between pair *i*, *j*:

8 $$\begin{array}{rcl}{x}_{ij} & = & {\rm{arccosh}}(\cosh \,{r}_{i}\,\cosh \,{r}_{j}-\,\sinh \,{r}_{i}\,\sinh \,{r}_{j}\,\cos \,{\rm{\Delta }}{\theta }_{ij})\\  &  & \approx {r}_{i}+{r}_{j}+2ln\,\sin ({\rm{\Delta }}{\theta }_{ij}/2)\\  &  & \approx {{r}}_{{\boldsymbol{i}}}+{{r}}_{{\boldsymbol{j}}}+2ln\,({\rm{\Delta }}{\theta }_{{ij}}/2)\,\end{array}$$

where

9 $${\rm{\Delta }}{\theta }_{ij}=\pi -|\pi -|{\theta }_{i}-{\theta }_{j}||$$

and *p*(*x*_*ij*_) is the Fermi-Dirac connection probability:

10 $${p}({{x}}_{{ij}})=\frac{1}{1+{{e}}^{\frac{1}{2{T}}({{x}}_{{ij}}-{R})}}$$

where *R* ~ ln*N*. The estimated radial coordinate of node *i* is based on its degree in the network (*k*_*i*_) via *r*_*i*_ ~ ln*N* − ln*k*_*i*_. Therefore, if node degrees are correlated in different layers so will be the radial coordinates^36^.

#### Proposed measures

Node degree or popularity plays an important role in defining the similarity measures and many of them are based on common neighbors and preferential attachment. The underlying principle behind preferential attachment is that new connections are mainly made to more popular nodes. However, Papadopoulos *et al*.^23^ showed that popularity is just one aspect of attractiveness, while similarity could be considered as another aspect. They developed a framework where new connections consider a trade-off between popularity and similarity.

We know that the degree distribution of many real networks follow power-law distribution. However, as it can be seen in As real multilayer networks, we consider six networks (see Table 1). The multilayer networks are converted to multiplex networks by assuming that all layers have the same number of nodes (the maximum number of nodes of all layers). Explanation of these networks is as follow:

**Table 1:** The topological features of six real multiplex networks. In the table, *i* is the number of layers, *N* is the number of nodes and *E* is the number of edges in each layer.

	i	N	|E|	〈k〉	S	H	Γ	T
Vicker	1	29	240	16.5	0.59	1.09	3.5	0.75
	2	29	126	8.69	0.31	1.27	3.5	0.85
	3	29	152	10.48	0.37	1.25	2.64	0.75
Lazega	1	71	717	20.19	0.28	1.16	3.5	0.75
	2	69	399	11.56	0.17	1.31	3.5	0.5
	3	71	726	20.45	0.29	1.16	3.5	0.8
CKM	1	215	480	2.23	0.02	1.61	3.04	0.55
	2	231	565	2.44	0.021	1.44	3.5	0.45
	3	227	504	2.22	0.019	1.33	3.5	0.35
CElegans	1	253	516	4.07	0.016	2.15	3.13	0.65
	2	260	888	6.83	0.026	1.78	3.35	0.85
	3	278	1703	12.25	0.044	1.67	2.79	0.85
Rattus	1	2035	3014	1.48	0.001	5.86	2.62	0.35
	2	1017	1093	1.07	0.002	3.99	2.14	0.25
SacchPomb	1	971	1686	1.73	0.003	2.72	2.92	0.9
	2	347	404	1.16	0.006	1.93	2.85	0.9
	3	2402	7502	3.12	0.002	3.92	2.68	0.25

〈*k*〉 represents the average degree, *S* is the density of each layer based on $({\boldsymbol{S}}=\frac{2{\boldsymbol{E}}}{{\boldsymbol{N}}({\boldsymbol{N}}-1)})$ and *H* is the degree heterogeneity obtained as $(H=\frac{\langle {k}^{2}\rangle }{{\langle k\rangle }^{2}})$, *T* is the temperature and γ is the power-law coefficient.

Table 1, the degree distribution of the multiplex networks with small size do not follow power-law distribution. The previous experimental results indicated that HP’s performance was better in the networks with power-law distribution and less good in those that does not obey power-law degree distribution^7^. The reason for that would be the way the nodes’ radial coordinates are calculated. Because one of the parameters which is considered in *HyperMap* method to estimate the radial coordinates is the power-law exponent of the network. Therefore, if the network does not have a power-law degree distribution, the link-prediction accuracy of HP decreases. In order to overcome this shortcoming and benefit the advantages of both popularity and similarity features of nodes, we proposed two approaches that are detailed in the following.

- **Weighted Common neighbors (WCN):** We generate a weighted version of CN that computes the weight of common neighbors considering the hyperbolic distance of them with the target node pairs. There are some studies about converting the original similarity measure to the weighted one, however it has been shown that that such conversion may reduce the prediction performance^37^. The pseudo-code of the proposed method is as follows:
  1. Approximate the hyperbolic coordinates of each node.
  2. Compute the matrix *H* of hyperbolic distance of the existing links in the network.
  3. *h* = average of *H*
  4. Γ_*ij*_ = list of common neighbors of node pair (*i*, *j*) in the test list of missing or spurious link prediction.
  5. for each *k* ∈ Γ_*ij*_
    if *H*(*i*, *j*) < *h*
    node pair (*i*, *k*) is a strong tie and has more weight
    *WCN*(*i*, *j*) = *WCN*(*i*, *j*) + 1 + 1/*H*(*i*, *k*);
    else
    node pair (*i*, *k*) is a weak tie and takes the weight as CN
    *WCN*(*i*, *j*) = *WCN*(*i*, *j*) + 1;
  6. Repeat step 5 for node pair (*k*, *j*)
  7. Sort all links in the test list in decreasing (for missing link prediction) or increasing (for spurious link prediction) order
- **Ranking CN and HP (CN-HP)**

This method benefits the advantages of both CN and HP measures. It uses a ranking method to combine the prediction given by both of these measures. In order to do that, one of the well-known classical rank aggregation methods, Borda’s method is used^38^. It is based on absolute positioning of the ranked elements rather than their relative rankings. A Borda score for each element is calculated based on the ranking of it in the aggregated list. For a set of full list *L* = [*L*_1_, *L*_2_, *L*_3_, …., *L*_*n*_], the Borda’s score for element *x* and list *L*_*k*_ is given by:

11 $${B}_{{L}_{i}(x)}=\{count(y)|{L}_{i}(y) < {L}_{i}(x){\rm{\& }}y\in {L}_{i}\}$$

and the total Borda’s score of element *x* is:

12 $$B(x)=\mathop{\sum }\limits_{i=1}^{n}{B}_{{L}_{i}(x)}$$

The advantage of Borda ranking method is that we can aggregate different kinds of measures with different categories and values and obtain a rank-based score. Also the computational complexity of this method is linear; however it does not satisfy the Condorcet criterion. In the proposed method, two lists of CN and HP scores for any node pair are constructed, and the final score for each node pair is computed by aggregating their ranking score using Borda method, i.e. in the case of missing/spurious link prediction the aggregated scores of CN and HP of all node pairs are computed based on Eq. (12) and are sorted descending/ascending. The top-*k* elements of the final list are the predicted links (*k* is the number of expected missing/spurious links).

#### Impact of layer relevance

In the case of HP as a similarity measure, the procedure of mapping each layer to its hyperbolic space can be done in different directions. One direction would be to jointly embed the different layers of a given multiplex and infer single radial and angular coordinates for each node. A second direction would be to aggregate the different layers using different operations such as those proposed in^39^, and then embed the aggregated network to infer single coordinates for nodes. Finally, a third direction would be to infer the node coordinates in each layer independently as considered here.

As it was mentioned above, we map each layer of each real multiplex to its hyperbolic space using the *HyperMap* method^6,24^. The method takes the network adjacency matrix and the network parameters *T*, *γ*. It then approximates the angular and radial coordinates of all nodes in the network. Parameter *γ* is the power law degree distribution exponent which is approximated separately for layers using the method introduced by Clauset *et al*.^40^, and *T* is the temperature. To estimate the values of *T*, the Nonuniform Popularity × Similarity Optimization *N-PSO* model is used^27^. The *N-PSO* model grows synthetic complex networks and it is equivalent to the hyperbolic *H*^2^ model. The inputs to this model are the final network size *N*, the average node degree *k*, power-law coefficient *γ* and the network parameters *T*. The *N-PSO* model is used to construct synthetic networks with the same size *N* and average degree *k* and power-law exponent *γ*, using different values for *T*. The estimated values of *T* are then the values that best match the degree distribution and average clustering between the layer and the corresponding synthetic network.

**Figure 1:**
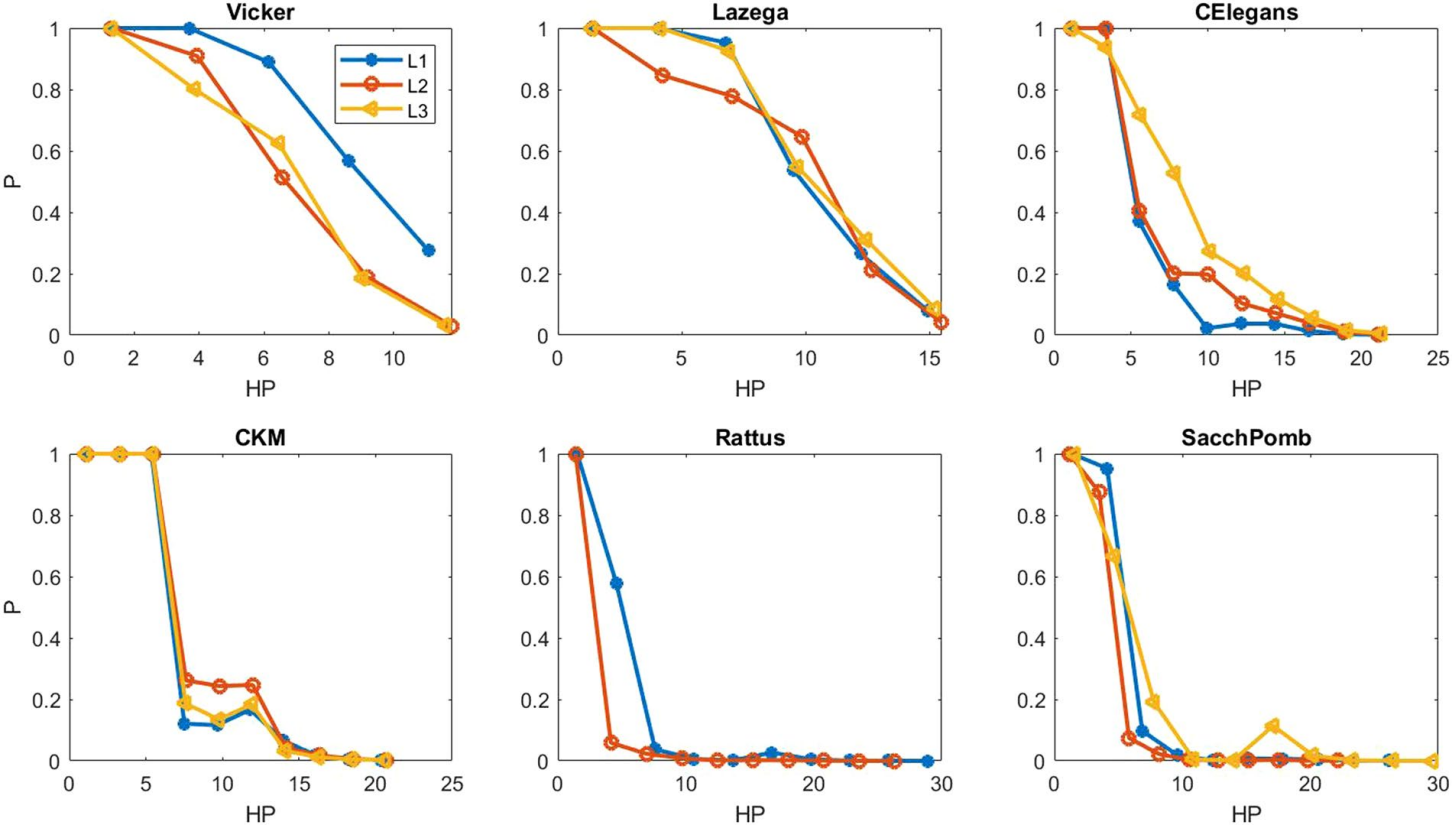
Probability of existing a link between node pairs based on their hyperbolic distance in different layers of multiplex networks denoted by L1, L2 and L3.

In order to test whether this measure can be a good one for the link prediction, we classify the hyperbolic distance of all node pairs, and compute the probability of the existence of a link between the pairs in each bin. To this end, first the hyperbolic distances of all node pairs are sorted in ascending order and divided to *k* bins. Bin *b*_*i*_ contains the node pairs with the hyperbolic distance in the range of [*d*_*i*_, *d*_*i*+1_]. Then, the probability *p*_*i*_ of having a link between the node pairs of each bin is computed based on the network topology. The results are shown in Fig. 1. As it is shown, the probability of existing a link between each node pairs decreases, while their hyperbolic distance increases. Two nodes have a smaller hyperbolic distance as much as they are popular or similar to each other, in this case the probability of existing a connection between them increases. Thus, this measure can be a candidate for the similarity score for the link prediction problem. It is worth noting that the behavior of different layers are almost the same in all multiplex networks, with being more similar in the bigger networks including Rattus and SacchPomb.

In order to employ the interlayer information, for node pair (*i*, *j*) in target layer *α*, we first calculate its similarity within each layer based on the proposed methods above. This enables us to compare prediction performance of the algorithms. In order to compare the prediction performance of the proposed prediction framework, we exploit different algorithms for quantifying the relevance between layers including link overlap, Pearson correlation, Spearman correlation and hyperbolic angular correlation^12^. The results show that the link overlap has the best effect on the link prediction performance. It is defined in the following.

- **Link Overlap (LO):** This measure identifies the ratio of common links in two layers, i.e. if *α* and *β* are two layers in a multiplex network, LO is the fraction of the same node pair that connects in both layers *α* and *β* and is defined as:

13 $${O}^{\alpha ,\beta }=\frac{{2\sum }_{i=1}^{N}{\sum }_{j > i\,}^{N}{A}_{ij}^{[\alpha ]}.{A}_{ij}^{[\beta ]}}{{\sum }_{i=1}^{N}{\sum }_{j > i\,}^{N}{A}_{ij}^{[\alpha ]}+{\sum }_{i=1}^{N}{\sum }_{j > i\,}^{N}{A}_{ij}^{[\beta ]}}$$

**Figure 2:**
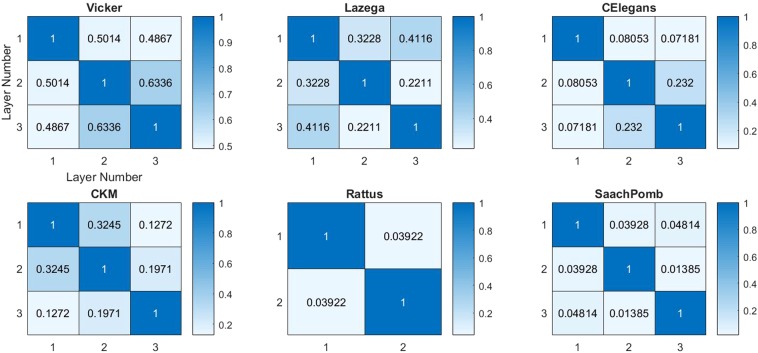
Link Overlap of different layers of six real multiplex networks.

Where *A*^[*α*]^ is the adjacency matrix of layer *α* that takes value of 0 for each disconnected node pair and 1 for each connected node pair, and *N* is the number of nodes. The value of *O*^*α*,*β*^ is in the range of [0, 1], where 0 indicates that the layers are completely irrelevant and 1 indicates that the layers are quite relevant. The similarity measure is defined as:

14 $$\forall i,j\in V:{S}_{ij}={s}_{ij}^{\alpha }+\mathop{\sum }\limits_{\beta =1}^{M}\,\eta {\mu }^{\alpha \beta }{s}_{ij}^{\beta }(\alpha \ne \beta )$$

where ${s}_{ij}^{\alpha }$ is the similarity index of target layer *α* and ${s}_{ij}^{\beta }$ is the similarity index of any other layer *β*. *μ*^*αβ*^ represents the correlation between layers *α* and *β* (link overlap), which can be explained as the weight of interlayer information involved from any layer *β* in link prediction in layer *α* and *η* is the tunable parameter. The correlations between different layers are shown in the Fig. 2. As it is shown, for all networks the link overlap correlation between different layers is positive. Furthermore, the highest layer relevance belongs to the Vicker network and the lower relevance belongs to larger and sparser networks. Our experiments show that LO is mostly consistent with other correlation metrics, but it has the most positive effect in the extent the interlayer information can improve the link prediction performance.

## Results

**Figure 3:**
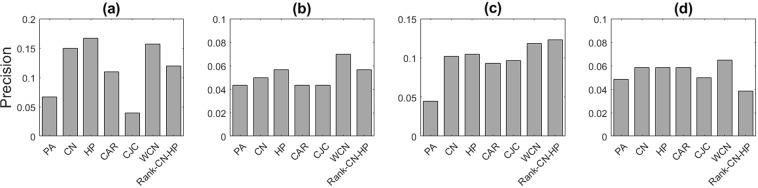
The missing link prediction performance of the synthetic networks based on *N-PSO* model, with (**a**) N = 500, m = 4, λ = 3, T = 0.3, (**b**) N = 500, m = 4, λ = 3, T = 0.6, (**c**) N = 1000, m = 4, λ = 3, T = 0.3, (**d**) N = 1000, m = 4, λ = 3, T = 0.6. Different similarity measures are used, including Preferential Attachment (PA), Common Neighbors (CN), Hyperbolic Distance (HP), CAR, CJC, Weighted Common Neighbors (WCN) and Rank-CN-HP. The results show the mean values over 20 independent experiments.

**Figure 4:**
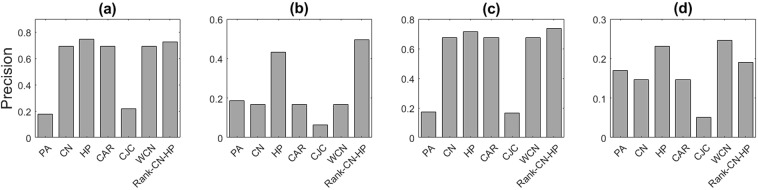
The spurious link prediction performance of the synthetic networks based on *N-PSO* model, with (**a**) N = 500, m = 4, λ = 3, T = 0.3, (**b**) N = 500, m = 4, λ = 3, T = 0.6, (**c**) N = 1000, m = 4, λ = 3, T = 0.3, (**d**) N = 1000, m = 4, λ = 3, T = 0.6. Different similarity measures are used, including Preferential Attachment (PA), Common Neighbors (CN), Hyperbolic Distance (HP), CAR, CJC, Weighted Common Neighbors (WCN) and Rank-CN-HP. The results show the mean values over 20 independent experiments.

We perform experiments on four single-layer synthetic networks to evaluate the similarity measures and six real multilayer networks to investigate the impact of interlayer information. The synthetic networks are evolved based on *N-PSO* model described above and their structural features and the precision of spurious and missing link prediction methods are presented in Figs 3 and 4. In the *N-PSO* model, the true node coordinates are generated for the networks. In the case of missing link prediction, we remove a fraction of edges using *k*-fold decomposition in each iteration and regenerate the node coordinates of the new network using the *Hypermap* method. Similarly, in the case of spurious link prediction, we add a fraction of nonexistent links to the network in each iteration and regenerate the node coordinates of the new network using the *Hypermap* method. There is no restriction in selecting the parameters for *N-PSO* model. It is preferred to generate networks with features that are near to real networks (large and sparse with power-law degree distribution) and temperature is chosen to be 0.3 and 0.6. Since the two parameters λ and *T* of *Hypermap* are set manually, so the approximation of hyperbolic coordinates is more accurate in synthetic networks.

Based on these reasons as it can be seen, in all cases the performance of hyperbolic distance (HP) is better than the other measures and the precision of the proposed methods (Weighted Common Neighbors (WCN) and Rank-HP-CN) is the highest in most cases. Thus, hyperbolic distance and its derived methods can be good choices as similarity measures for link prediction.

As real multilayer networks, we consider six networks (see Table 1). The multilayer networks are converted to multiplex networks by assuming that all layers have the same number of nodes (the maximum number of nodes of all layers). Explanation of these networks is as follow:

1. Vicker^41^: It is a 3 layer multiplex network with 29 nodes representing the students of a school in Australia. The layers are defined as the contact relationship, co-working and best friends.
2. Lazega^42,43^: This multilayer network represents the partnership of corporate law between associates and partners. The layers correspond to co-working, friendship and advice relationship.
3. CKM^44^: This multilayer network represents the interactions between physicians. It contains 3 layers that correspond to friendship, discussion and asking for advice.
4. CElegans^45,46^: It is a biological multilayer network in which nodes represent neurons and layers correspond to chemical monadic, chemical polyadic and electric interactions.
5. Rattus^47,48^: It is a multiplex genetic and protein interactions network of the Rattus Norvegicus. It contains two main layers of physical association and direct interaction.
6. SacchPomb^47,48^: It is a multiplex genetic and protein interactions network of the Saccharomyces Pombe. It includes three kinds of relationships, including direct interaction, colocalization and physical association.

The experimental results of the proposed link prediction methods on six real networks is presented in this section. For each multiplex network, the layer with the most density is chosen as the target layer. In the case of missing link prediction, 15% of links in the target layer are considered to be hidden and based on k-fold decomposition method, the performance of the similarity measures are examined over 20 independent experiments. For spurious link prediction, random links are added to the network and the performance of the similarity measures are examined over 20 independent experiments. In order to evaluate the impact of employing the layer relevance and the extra information of other layers, we separately study the performance of the algorithms on single-layer (when only information of the target layer is considered) and multiplex (when inter-layer information is also considered) fashions. We employ the layer correlation based on link overlap and compute the similarity measures based on Eq. (14).

**Figure 5:**
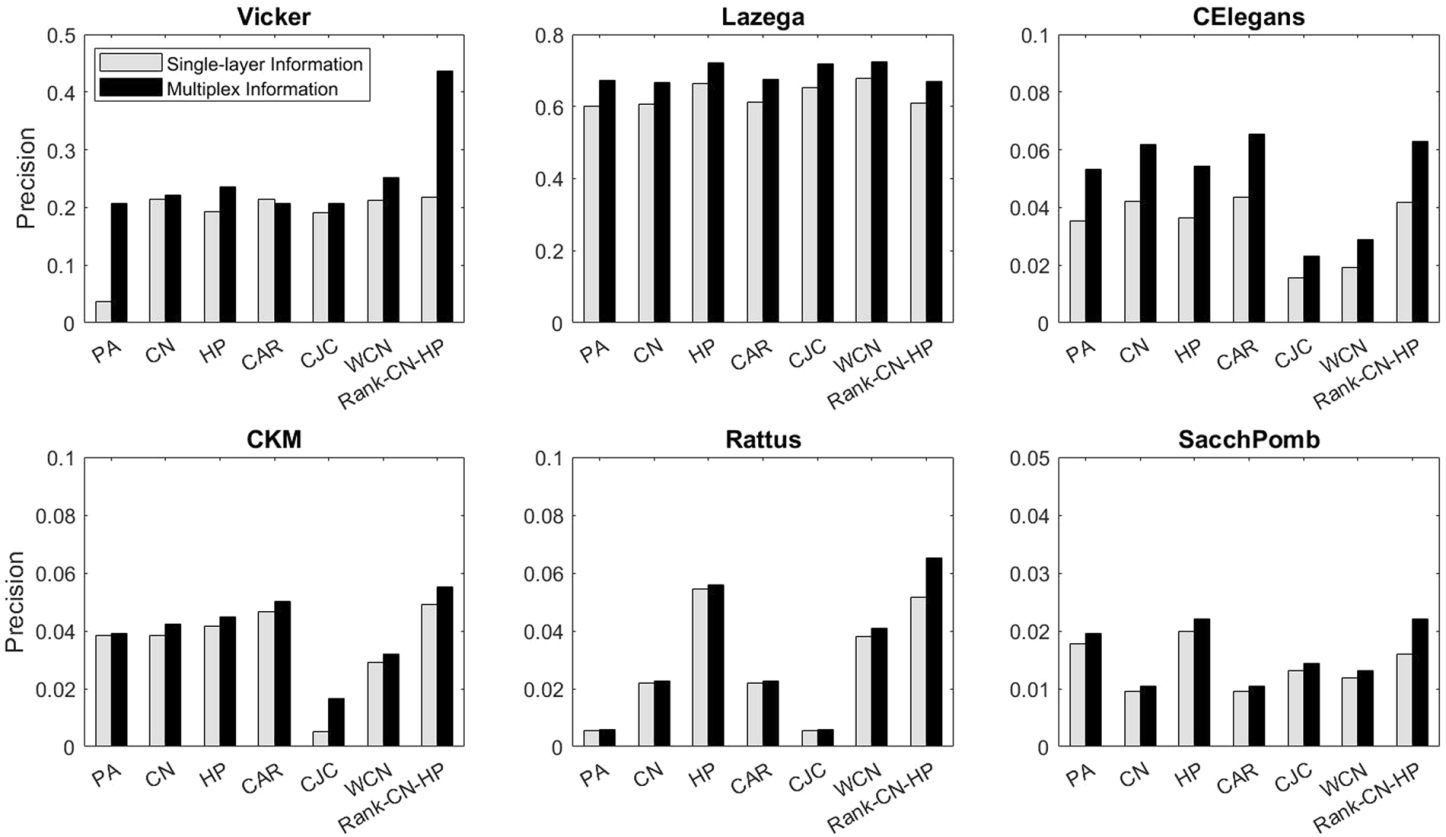
The missing link prediction performance of the multiplex networks based on different similarity measures. The results are based on the mean values over 20 independent experiments. ‘Single-Layer Information” corresponds to the case when only intralayer information of the target layer is considered. “Multiplex Information” corresponds to the case when both intralayer information of the target layer and interlayer information of other layers are considered.

Figure 5 shows the precision of missing link prediction of different similarity measures. For each measure there are two bars. The left bar shows the performance of the similarity measure while considering only the intralayer information of the target layer and the right bar is the performance of the similarity measure while using both intralayer and interlayer information. As it can be seen, in all cases incorporating the interlayer information improves performance of the missing link prediction and this is more pronounced in CElegans. The proposed similarity measures Rank-CN-HP has the best performance in most cases.

**Figure 6:**
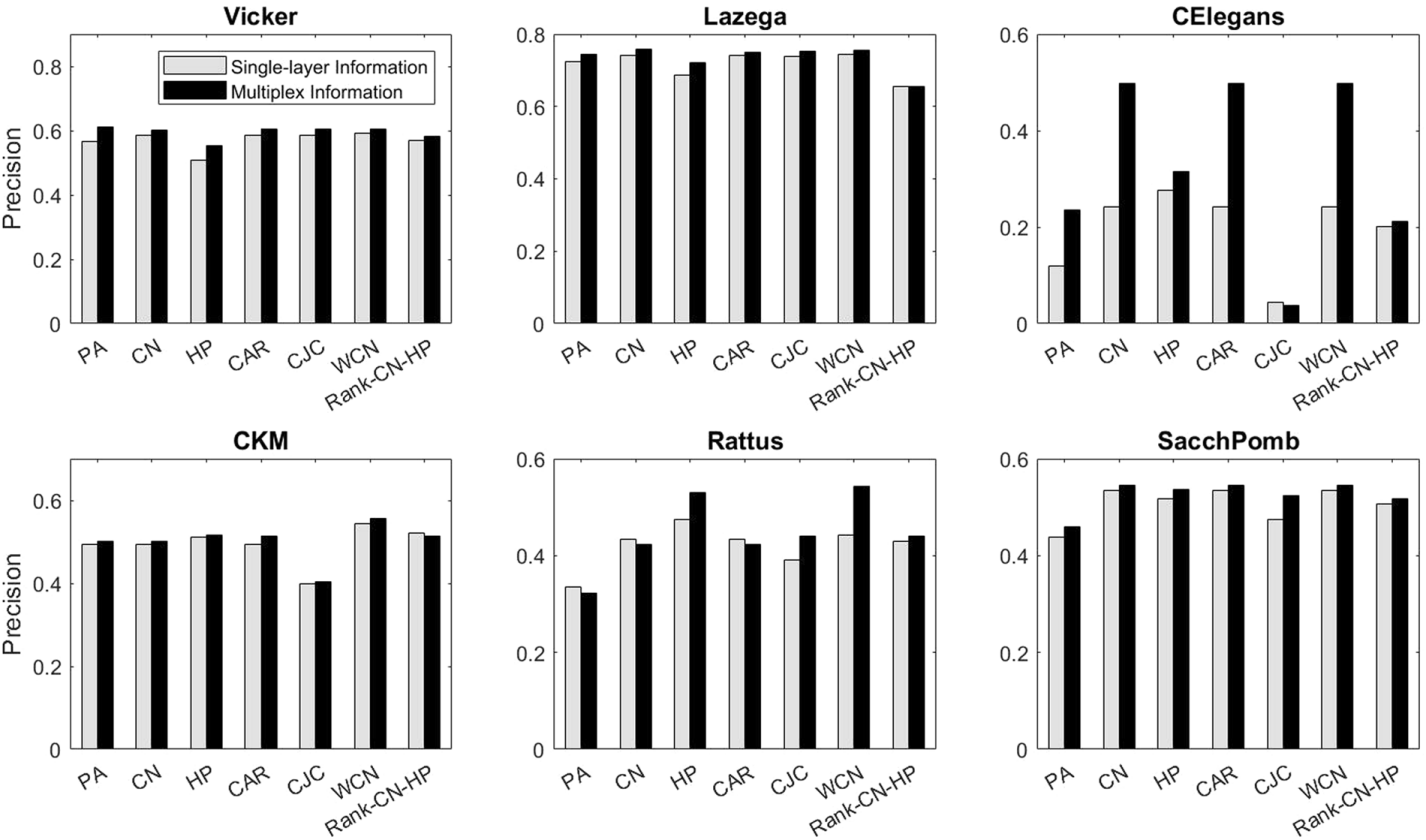
The spurious link prediction performance of the multiplex networks based on different similarity measures. The results are based on the mean values over 20 independent experiments. ‘Single-Layer Information” corresponds to the case when only intralayer information of the target layer is considered. “Multiplex Information” corresponds to the case when both intralayer information of the target layer and interlayer information of other layers are considered.

Figure 6 shows the performance of the algorithms on spurious link prediction. For this problem, we also consider the cases when only intralayer information of the target layer is considered and when both intralayer and interlayer information are considered. As it can be seen, in most cases, including the interlayer information in the prediction process improves the performance. Furthermore, predictions based on PA similarity measures have the worst performance in most cases. In contrast to the missing link prediction, in this case Rank-CN-HP is not better than CN or HP in some of the networks, but WCN has the best performance in all multiplex networks. Our experiments show that in small networks, in the case of HP, the approximated radial coordinates of nodes in hyperbolic space for both true positive (correctly predicted) links and false negative links are almost in the same range. But the average degree of true positive links is significantly higher than the false negatives. It means that the radial coordinates of nodes which corresponds to their popularity are not precisely approximated, since the degree distribution of the target layers do not obey the power-law. HP mostly represents the similarity of node pairs, and thus it is not expected in most cases to have high performance. Therefore, combining this similarity measure with CN in different ways help to overcome the shortcoming of HP in covering the popularity attribute of each node. On the other hand, in large networks and especially in those with scale-free degree distribution, approximating the underlying hyperbolic geometry is more precise, but these networks are mostly sparse and similarity measures such as CN, CAR and CJC may not be quite successful in link prediction. Thus, in such cases combining the popularity-based measures with HP can improve the link prediction. The Rank-CN-HP and WCN methods both use CN as the popularity factor, and HP as the similarity factor. The difference is that in Rank-CN-HP the proposed similarity measure uses CN and HP independently, i.e. these two measures are first computed independently for each node pair, and then ranked based on Borda rank aggregating algorithm to achieve the final score that considers both CN and HP with the same weight. Whereas in the WCN method, we compute HP-distance for the common neighbors of each node pair and compare them with a threshold. If the HP-distance is less than the threshold, that node pair is assumed to have a strong tie, i.e. they are more similar to each other, and thus a fraction of HP-distance is added to the weight of that common neighbor; otherwise it is computed as the original CN. Therefore, in the WCN method the two similarity measures are dependent to each other.

## Discussion

In this work, two novel methods based on the hyperbolic geometry of the multiplex networks are proposed to discover spurious and missing links in multiplex networks. The hyperbolic underlying of complex networks considers two parameters of popularity and similarity of nodes that both play important role in link prediction problem. Since the common local similarity measures mostly consider only the node degree (popularity), we suggest to enhance their predictability by adding the similarity feature to them. As we can see, in the case of missing link prediction specifically in social networks, each node is more likely to connect to nodes with similar features (his friends) as well as popular nodes (influencers). Another hypothesis is that interlayer relevance can be helpful in link prediction. Based on this hypothesis, recently a new method was proposed that considered the existing similarity measures in both target layer and other layers and combined the similarity measures via a correlation metric (Link Overlap) and obtained a multiplex-based similarity measure for spurious link prediction^21^. Based on this research, new measures are proposed based on the hyperbolic geometry of the network. First, a number of existing similarity measures which are widely used for the link prediction are chosen and then new measures are proposed to solve the spurious and missing link prediction problem. Our experimental results on four synthetic networks and six real-world multiplex networks shows that the new proposed measures outperform in all cases and also incorporating the interlayer information can improve the prediction performance compared with the case that only intralayer information is considered.
